# Low level of community readiness prevails in rural northwest Ethiopia for the promotion of institutional delivery

**DOI:** 10.11604/pamj.2021.38.281.27300

**Published:** 2021-03-17

**Authors:** Adane Nigusie, Telake Azale, Mezgebu Yitayal, Lemma Derseh

**Affiliations:** 1 Department of Health Education and Behavioral Sciences, Institute of Public Health, College of Medicine and Health Sciences, University of Gondar, Gondar, Ethiopia,; 2 Department of Health Systems and Policy, Institute of Public Health, College of Medicine and Health Sciences, University of Gondar, Gondar, Ethiopia,; 3 Department of Epidemiology and Biostatics, Institute of Public Health, College of Medicine and Health Sciences, University of Gondar, Gondar, Ethiopia

**Keywords:** Community level of readiness, community readiness model, institutional delivery

## Abstract

**Introduction:**

the health benefits of institutional delivery with the support of skilled professional are one of the indicators of maternal health status which have an impact on the health of women and new coming generation. Despite these benefits, many pregnant women in Ethiopia are not actively bringing delivery at health facility. This study was aimed at determining the readiness level of community for promoting child birth at health facility.

**Methods:**

a population-based cross-sectional study was conducted. We interviewed 96 key informants using a semi-structured questionnaire adapted from the community readiness assessment model and translated to Amharic language. The key informants were purposively selected in consultation with the district health office to represent the community. The interviews were transcribed verbatim and survey scores were matched with the readiness stage of 1 of the 9 for the five dimensions using the assessment guidelines.

**Results:**

this study placed nine kebeles at stage 3 (vague awareness), which indicates the need for more institutional delivery service strategy programming; efforts of the community were not focused and low leadership concern and one kebele was in stage 2 (denial/resistance). Six kebeles were placed at high level of readiness i.e. in stage 7 (stabilization), indicating actions are sustained by the local managers or opinion leaders.

**Conclusion:**

evidence derived from the present study can be used to match intervention tactics for promoting health facility child birth service utilization to communities based on their level of readiness.

## Introduction

Worldwide, an enormous amount of women have died as a result of maternal causes in 25 years between 1990 and 2015 [1]. Developing countries were taking the majority segment of maternal health complications and deaths. Three-fourth of the deaths was due to preventable direct obstetric complications [2-6]. Making child birth at health facility ensures safe child birth, reduces actual and potential complication and maternal death; and improves the survival of mothers and newborns. However, most deliveries in developing countries occur at home without skilled birth attendants [1, 7].

In sub-Saharan Africa, the death of woman from preventable complication of pregnancy and child birth were high as compared to in the developed regions [8]. Even though the maternal mortality in Ethiopia shows a declining trend from 2011 Ethiopian Demographic and Health Survey (EDHS), it is still high, accounting for 412 deaths per 100,000 live births [9]. Since the setup of health care system in developing countries were not well and equitable, women chances of accessing and receiving institutional delivery care were influenced by the health care seeking behavior of a woman. The burden of maternal death could be unbalanced due to mothers with low health care seeking behavior regarding institutional delivery.

A community-based intervention must ensure the local level need, the dynamics of the community and willingness to participate in the promotion events [10]. Those researchers involving community mobilization for the intervention activity work to evaluate the feasibility and usefulness of various adapted community based interventions, so that they must determine meaningful ways to gauge the readiness level of each community for promotion and prevention activities. To confirm a more successful intervention study evaluating the readiness level of community to participate in the promotion events adapting an intervention which holds the necessary potential were a crucial step [11-13]. Therefore, promoting health facility child birth service utilization is a priority adjenda in Ethiopia. The participation of any concerned organization in an intervention was the primary extent of adoption. This adoption can also involve an assessment of the readiness level of community [14]. We used the Community Readiness assessment Model (CRM) and conducted interviews of key informants to determine the readiness level of each community [15]. To our knowledge, no study has applied the CRM to the promotion of institutional delivery service utilization.

## Methods

**Study setting, approach and participant selection:** a cross-sectional survey of key informants was conducted on the readiness level of communities to interact with the promotion of health facility child birth service utilization programs in rural areas of Central Gondar Zone from August to December 2019. The Central Gondar zone is found in Amhara National Regional State (ANRS) and its capital city, Gondar is found 180Kms far from Bahir Dar, the regional capital city, and 727Kms far from, the capital city of Ethiopia. Conferring to the 2019 Central Gondar zone health department report, there have been 14 districts (2 urban and 12 rural), 75 health centers and 9 hospitals within the zone. We selected 15 kebeles from 2 districts (seven kebeles from Wogera and eight from Dembiya) for the readiness assessment. Following the CRM working protocol 6-8 key participants from each kebele with a total of 96 key informants were purposively selected in consultation with the district health office.

**Data collection tool and procedure:** to determine the readiness level of community to have an interaction within the promotion of health facility child birth service utilization efforts, we adapted the CRM assessment tool i.e. a semi-structured key informant interview guidelines were developed and pilot tested by following the protocol of CRM [16]. According to the CRM working protocol interviewing 6-8 key participants is recommended to know the real problem of the community since the question asked for the key participant is to speak regarding the broader public perspective. Therefore, we have conducted a face to face base line readiness interviews on 96 key participants before intervention. The interview was conducted at the center of each kebele; a place where most the participant was comfortable. Yet, usually within qualitative research, researchers aim to succeed in a degree of ‘speculative saturation’ where by no novel ideas are anticipated to be gained by guiding more interviews [17]. In this sense the research is more driven by the information than by any preconceived views of the community situation that the researcher may have.

This research sought to attain ‘speculative saturation’ with the suggested number of key informants. ‘Speculative saturation’ at the community level was assessed iteratively using thematic analysis of every transcript in sequence to detect whether any additional themes were presented compared with the previous transcript. Thus saturation was reached when little new information or themes were contributed by the last transcript [17]. The interview guide had five sections corresponding to the model´s dimensions: the knowledge of the community´s on the health problem and efforts; efforts of the community; the leadership within the community; the community´s attitude; and the resources available to support the issue [13, 15, 16, 18, 19].The engagement of the community with the promotion of health facility child birth service utilization was considered to be a behavior with various factors influencing whether individuals perform them, therefore the interview questions addressed the engagement of the community with the promotion of institutional delivery service utilization. In addition to taking field notes on face-to-face discussions, an audio-recorder was used for participants who gave consent to be recorded. On average, a 1h and 25 minute's time was given for each KII.

**Trustworthiness:** the interview guide were pilot tested in a place where out of the study area but with similar socio demographic characteristic. The principal investigator (AN) has been in the field for a long time and has been involved in data collection activities, which helped to capture the reality of those being studied. In addition, daily base debriefing is conducted with research assistance. Collected data were shared with all co-author, who gave critical comments and suggestions. Verbatim transcriptions of the data were taken. The data collected were triangulated with those from field notes and observations during the analysis to increase validity and credibility.

**Community readiness model:** this model was a community based model developed in 1994 at Colorado State University with the aim of building the capacity of communities by integrating a community´s culture, resources, and level of readiness to more effectively address a specified health issue. It uses key informant interviews to assess the readiness level of a community to address local health issues [16]. Even though the CRM was developed for the prevention of alcohol/drug abuse, it has been utilized for the prevention of HIV/AIDS, violence, and smoking [15, 19-21]. It can even be utilized within the promotion of various health programmes. The CRM may be tailored to a specific issue, relies on local experts, and provides individual dimension scores that´s why we chose it [16]. CRM has 4 properties: 1) In the process of addressing a public health problem communities are at diverse level of readiness; 2) the level of readiness stage may be correctly measured; 3) to plan, apply, sustain, and advance effective programs communities pass all the stages; and 4) interventions which are needed to maneuver communities through the stages differ by stage of readiness [13]. The CRM includes an interviewer-administered survey for key informants which will be tailored to a problem. The survey had construct validity and high inter-rater consistency [22]. The CRM had two phases i.e. the assessment phase and application phase.

The assessment phase had four steps: (1) problem identification; (2) community description with regard to the issue; (3) conducting key respondent interview; and (4) scoring the responses of the interviewees. The second phase of community readiness is that the appliance phase and it had the last word steps: (5) developing community-specific strategies supported the stage of readiness as determined within the assessment phase and (6) implementing those strategies. To intellectualize a readiness of community, nine stages were defined as a part of the CRM and include: (1) no awareness: the community accepts the behavior as normative; (2) denial: belief that the problem doesn't exist within the community; (3) vague awareness: problem recognition, however no plans to require action; (4-5) awareness of the issue and preparing to require action; (6) initiation: indicates a program is being implemented; and (7-9) full awareness/implementation of programs, including effective training and evaluation that results in a high level of community ownership. Based on the scoring of the interview responses (step 4) we determined which of the nine stages best described the readiness level of the community. These stages focused on community behavior change instead of individual behavior change [16]. This article provided results on the primary four steps as an appropriate baseline before an intervention was developed within the community, still as indicated proposed activities to start the 5^th^ step of developing community-specific strategies.

**Community, study population, and interview process:** ninety six key respondents living and dealing in 15 kebeles from East Dembiya and Wogera districts were selected for the CRM assessment using questionnaires developed to assess promotion of health facility child birth service utilization (prevention of home delivery). These individuals were chosen based on their social roles within the community focusing on the knowledge regarding the promotion of health facility child birth service utilization (prevention of home delivery), the involvement in community and government organizations, as well as representativeness for the Central Gondar zone districts. The questions were developed to decide the community level of readiness for every of the subsequent dimensions: (1) knowledge of issue; (2) efforts of the community and knowledge on the efforts; (3) leadership; (4) attitude of community; and (5) resources.

Additional questions pertaining to home delivery prevention and health service utilization behaviors of the community were included, but were not part of the CRM assessment. The responses to those additional questions provide important community information to be used during the event and implementation of community-specific strategies (application phase). The protocol for the study and the questionnaires (written in English first, and then translated to Amharic language) were reviewed and approved by the University of Gondar Institutional Review Board (Protocol R. No: -O/V/P/RCS/05/1048/2019; Date: -04 March 2019). All interviews were conducted from August 28 and November 30/ 2019 and Key respondent interviews were completed in a place where comfortable for the respondents and were audio recorded. Informed written consent was obtained prior to conducting the interviews. These recordings were transcribed and translated from Amharic into English for scoring purposes.

**CRM scores and analysis:** five readiness score were given for each of the completed interview, one for every dimension, using dimension-specific rating scales. The given scores were then averaged by each dimension across all key informants to run a summary of dimension readiness scores for the community. Each dimension score corresponded to a stage of readiness, together with an overall average readiness score that was calculated from the five dimension scores. Two trained CRM scorers were involved in the scoring process of the finding for the entire interview and there were no significant deviances to the scoring protocol. For each response item we had determined the frequency and proportion. Then we analyzed categorical data. Missing data had been deleted in the event of item non response. The median and inter quartile ranges for ordinal data had been calculated. Following procedures described by Plested and Edwards [23], we obtained the mean, standard deviation, and range for each dimension score and the stage of readiness score [23].

The numerical data were supported by the tallied responses from open-ended questions. Key Informants based their responses on their experiences in the respective kebele of the study area. The response of the key respondents for the open-ended questions of the CRM was transcribed properly. Then, the transcribed data were translated into English. The data were uploaded into an ATLAS.ti 8 software program for coding purposes. Two individuals performed the coding separately. Using an ATLAS.ti 8 training manual, the analysis was done using the four phases of theme development: 1. familiarization with the data, 2. re-visit research objectives, 3. develop a framework, and 4. identify patterns and connections. First, open codes were created by reading the transcripts line by line, i.e., quotation was created; and then, words with similar meanings were grouped into categories. In the next step, we performed selective coding and further categorized relevant codes to form themes. Noticeable themes were then further categorized into sub-themes. In such instances, we allocated their responses to the kebele from which they were recruited for the study. All numerical analyses were conducted using SPSS version 23.

**Ethics approval and consent to participate:** this study was done in accordance with the Declaration of Helsinki. The Ethical approval was gotten from the institutionalized review board, institute of public health, university of Gondar. Official letter that explains the objectives of the study was written to the respected Central Gondar Zone administration and zonal health office. The zonal administration and zonal health office in turn were wrote a letter to study kebeles for cooperation respectively. The objectives and the benefits of the study were explained for the study subjects. We obtained written informed consent from the participant. On top of that, figure print was taken from those participants who did not able to read and write, and this was approved by the ethical committee. The right of the participants to withdraw from the study whenever they want to do so was respected. Anonymous questioner was used to protect the identity and confidentiality of the information obtains from individual participants.

## Results

**Characteristics of the participants:** ninety six key respondents completed the CRM questionnaires on the issues of promotion of institutional delivery service utilization/prevention of home delivery. The study revealed that forty seven (49%) of respondents were females and their age was ranged from the year of 20 to 60 and most of the participants were in the age of 25 to 34 years. These participants were chosen to be key informants supported their various areas of experience. A minimum of six key informants were interviewed from each kebele. All respondents were used in the scoring process of the level of readiness. The key informants were represented varied positions in the kebeles; the most common were different association heads in the kebeles (31.3%), kebele Administrators (29.2%), Health Development Army leaders (24%) and Health Extension Workers (15.6%) (Table 1).

**Table 1:** socio-demographic characteristics of study participants at Central Gondar Zone, Northwest Ethiopia, 2019 (n=96)

S.N	Characteristics		Frequency (%)
1	Sex	Male	47(49%)
		Female	49(51%)
2	Educational status	Illiterate	10(10.4%)
		Can read and write	17(17.7%)
		Primary(grade 1-8^th^)	39(40.6%)
		Secondary and above	30(31.3%)
3	Age	18-24years	4(4.2%)
		25-34years	40(41.7%)
		35-44years	33(34.4%)
		>=45years	19(19.8%)
4	Responsibility	Kebele administrators	28(29.2%)
		Different associations	30(31.3%)
		HEW	15(15.6%)
		HDA leaders	23(24%)
5	How long lived in the kebele	<=5years	9(9.4%)
		>5years	87(90.6%)

**Figure 1:**
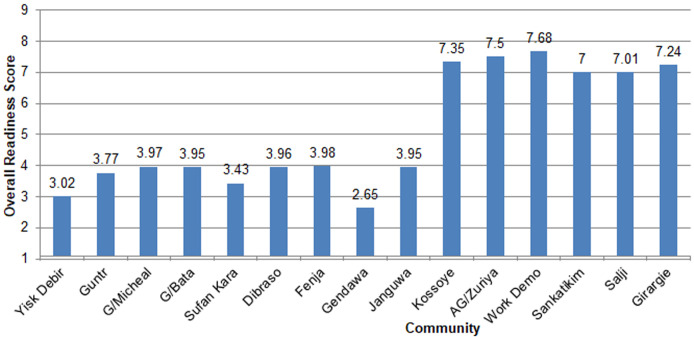
over all readiness score out of nine point of scale in Central Gondar Zone, Northwest Ethiopia, 2019

**Figure 2:**
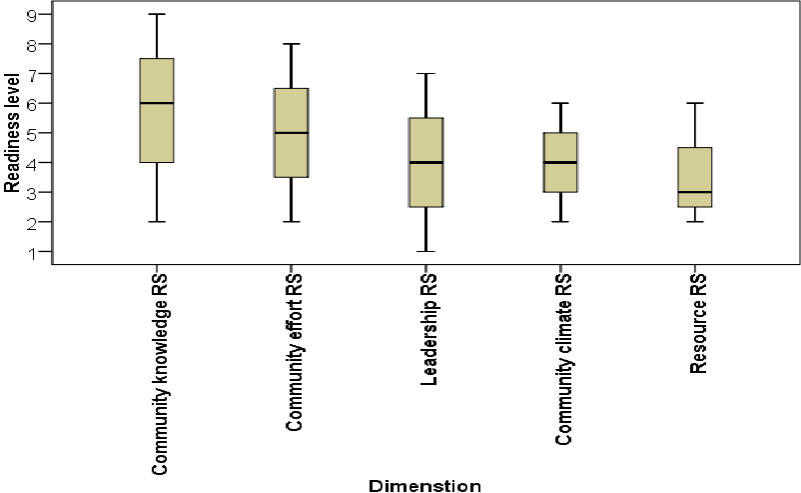
community readiness results that show the full range of scores across each dimension (mean plus minimum and maximum scores) in Central Gondar Zone, Northwest Ethiopia, 2019

**Readiness of the community on the promotion of institutional delivery service:** the results of community readiness assessment on the promotion of institutional delivery service are provided in Table 2. The study revealed that overall readiness score was 4.6 ± 2.028 standard deviation (SD), indicating that the readiness level of community is at the preplanning stage. With regard to specific readiness dimensions, knowledge of the community regarding the health problem received a score of 6.67 ± 2.225 SD, the initiation stage. Efforts of the community and knowledge of efforts received a score of 5 ± 2.236 SD, the preparation stage. Leadership scored a 4 ± 2.154 SD, which falls into the preplanning stage. Attitude of community is also was in the preplanning stage with a score of 4 ± 1.732 SD, preplanning stage. Resources received a score of 3.73 ± 1.58 SD, the vague awareness phase (Table 2). The study also revealed that the overall scores ranged from 2.65 to 7.68 out of nine points. Among the total of 15 communities eight scored at stage of vague awareness, one scored at stage of denial/resistance and six scored at stage of stabilization (Figure 1). The mean scores for each dimension ranged from 3.76 to 6.67. The overall stage of readiness score was 4.6, identifying the 15 communities as being in the CRM stage of “preplanning stage” (Figure 2).

**Table 2:** community readiness scores of the CRM dimensions for promoting institutional delivery services in Central Gondar Zone, Northwest Ethiopia, 2019

S.N	Dimension	Average Readiness level ±SD(Readiness level)
1	Community knowledge	6.67±2.225 (Initiation)
2	Community effort	5±2.236 (Preparation)
3	Leadership	4±2.154 (Preplanning)
4	Attitude of community	4±1.732 (Preplanning)
5	Resource	3.73±1.58 (Vague awareness)
Over all readiness		4.6±2.028 (Preplanning)

**Home delivery prevention behaviors within the community:** in addition to asking questions based on the CRM, the interviewers were allowed to collect information to help develop community-driven strategies for implementing specific activities focused on reducing home delivery/improving institutional delivery service utilization. When asked regarding the misconceptions held by the community members about institutional delivery, key respondents stated that fear of bad behavior of health workers, distressing of the baby, exchange of baby, and blood drawing; and fear that their body might be touched by health workers which makes them ashamed of being touched, and the waiting home may not be enough for families.

In regards to current efforts occurring to improve these issues, key respondents stated that there were efforts available and the community was familiar with the efforts but they were not actively involved in the activity, there were no enough resources available. The efforts which were being made in the area included educational programs to prevent home delivery, availability of traditional ambulance, supportive supervision by Health Extension Workers (HEWs) and Health Development Army (HDAs). Incorrect information on the efforts held by the community members as stated by the key respondents was relating HDAs with unique benefit, suspicious towards the work of HDAs and fear of the waiting home at health facility. When asked regarding the concern of community leaders, the key respondents state that most of the leaders were not actively involved and they thought that this was the task of the HEWs and female HDAs. The leaders were occupied by other many administrative tasks and home delivery prevention was not a priority issue.

The key respondents stated that most of the community members did not prioritize the home delivery prevention; most of the community members wanted to visits health facility for the delivery service after they tried home activity. There were conditions in which community members think home delivery should be tolerated; there is a belief that it is normal thing, let´s wait until animals get in, let´s celebrate for the environment with coffee she may deliver in between, let´s wait until the sun raise she may deliver in between and let´s wait until the sun is shine she may deliver in between. As stated by the key respondents the main problems faced in home delivery prevention were poor road infrastructure, long distance, cultural belief, family influence and lack of resources.

**Figure 3:**
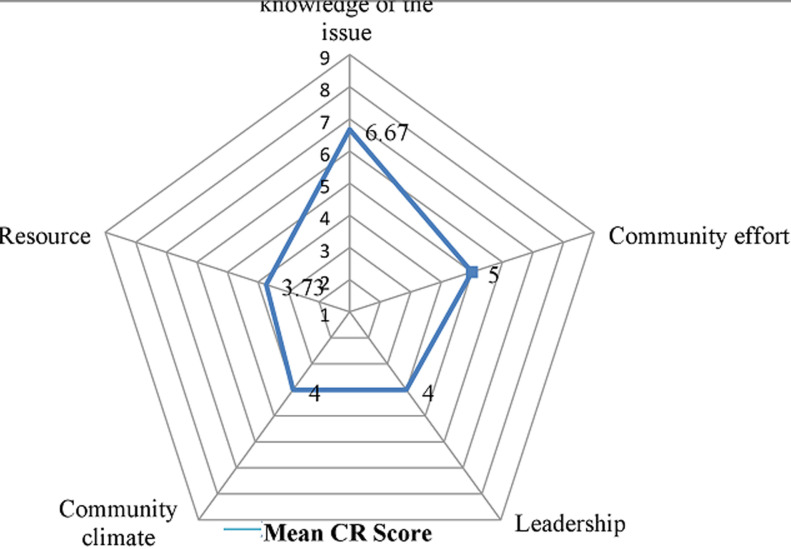
average community level of readiness (CR) score per measurement for the fifteen communities in Central Gondar Zone, Northwest Ethiopia, 2019

**The readiness of community to address an issue on five key dimensions of CRM:** the average community level of readiness (CR) score for all the five dimension per measurement for the fifteen communities were indicated by the cobweb chart from stage 1 (vague awareness) to stage 9 (ownership) (Figure 3).

**Dimension A: knowledge about the problem:** the study revealed that 55 (57.3%) of the key informants responded that community members had a close knowledge on the promotion of institutional delivery service utilization, as shown by the quotes below:

*“Community members are there, who participate continuously in meetings and make frequent contacts, which they're cognizant but there are also people that don´t have the knowledge there are still many folks that don´t want to administer birth at health centers because of shyness“...(Female HDA leader)*

*“Females who are birthing for the first time don´t want to bare their body, they feel ashamed since they don´t have experiences”...(Female HDA leader)*

Fifty (52.1%) of the key informants responded that the community members´ had awareness regarding the advantages of institutional delivery. Fifty five (57.3%) of key informants thought that information about health facility child birth service utilization was readily available, while only 41(43%) of the key informants ensured the community members utilized the information available to them. The foremost commonly cited source of information on health facility child birth service utilization was HEWs, HDAs, the media (radio, newspaper) and other health care providers from the health center. Health facility child birth service utilization information was possibility to be obtained through HEW, HDA, other health care providers, and other public gatherings like churches, as shown by the quotes below:

*“Birthing at health facility could protect from many diseases which can´t at home; the material used at the health facility is clean, no communicable disease at health facility, at home most of the time there is a trend of sharing in utilizing materials. The community heard this all from HDAs, HEWs, 1 to 5 HDAs leaders, 1 to 30 HDAs leaders”...(Health extension worker)*

*“The community were not utilizes the information fully. There is poor utilization of the information by most of the community. There is part of the community who says I shall give birth at home due to fear of the transportation i.e. once the ambulance took the pregnant women for the child birth service, it does not bring back the mother to her home ”...(Health extension worker)*

**Dimension B: efforts of the community and knowledge of the community about efforts:** regarding the knowledge of the community members on the efforts, 69 (71.88%) of Key informants were aware of health facility child birth service utilization programs that were no longer in existence. The reasons cited for not giving birth at health facility included a lack of income, lack of waiting home, fear of the bad behavior of health professionals, and influence of families especially mothers in-law. Forty four (45.8%) of the key respondents reported that the community members were aware of current programs for institutional delivery service utilization. Only 19 (19.8%) of the key respondents stated that those programs had been active for less than a year. Thirty five (36.5%) of the key respondents stated that the community members had a concern about health facility child birth service utilization. The median rating for concern of community members about health facility child birth service utilization levels among communities was two. The median rating for awareness of community members on the existence of programs to promote institutional delivery in the community was three. The most commonly cited strengths of the programs were house to house visiting of pregnant women by the volunteers. The most frequently reported weakness was lack of the motivation of the community in order to take part on the promotion of institutional delivery care seeking behavior. In addition to this, most of the community members externalized every promotional activity for others.

*“Most of the community knows the efforts undertaken to prevent home delivery (8/10) but the participation of the community is not as such good and so that the efforts of the community could be around 75% (7/10) .”...(HEWs)*

*“Half of the community members did not participate on the activity to prevent home delivery. So I could score no concern (5/10) .” (Female HDA leaders)*

*“There is cultural and social influence, the older people make influences not to go to health center, some people go to witchcrafts, everyone is not equal in terms of awareness and there is men´s influence because even when they see other pregnant women giving birth at health center well, they resist you. Lack of motivation of the community on the promotion of institutional delivery care seeking behavior´ was the weakness of the program” (Head of kebele female affairs)*

*“Enabling of knowledge creation via house to house visiting of pregnant women by the volunteers for the large community. Follow-up by calling phone, House to house visiting, registration of pregnant women, advising, Availability of Ambulance was the strength of the program” (Head of kebele youth affairs)*

**Dimension C: leadership:** only few 12 (12.5%) of key informants were aware of leaders who were working to promote health facility child birth service utilization in their communities. The types of leaders that were most frequently mentioned by the key informants were Kebele administrators (71.9%). Some key informants mentioned groups involved with kebele wide initiatives for promoting health facility child birth service utilization. Conferring to the key informants, many leaders were not promoting the issue i.e. health facility child birth service utilization the median rating for the perceived concern of community leaders for improving health facility child birth service utilization levels in their community was 1 ± 0.696.

*“The leadership was not actively involved in the prevention of home delivery except HEW and female HDA. The only thing what the leaders did was that information dissemination in the meeting (message transferring, speaking for the community) using the opportunities where the community has a gathering event in every aspects. Almost all the leadership had not attention for the prevention of home delivery. Only HEWs and female HDAs actively involved but other leaders including school leaders and others were not involved. So I could score it very low1/10 ” (Female HDA leaders)*

**Figure 4:**
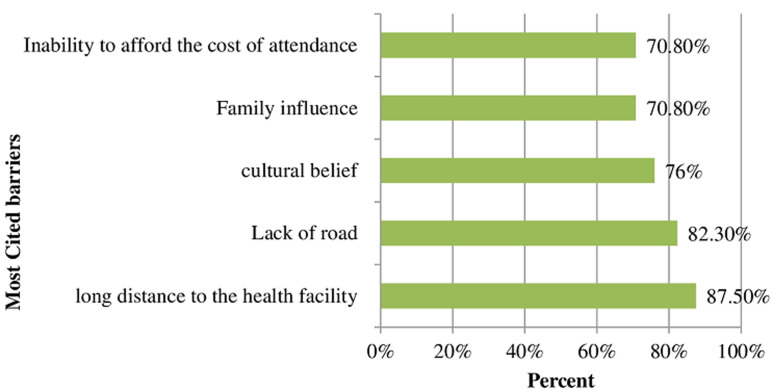
most commonly cited barriers for institutional delivery in Central Gondar Zone, Northwest Ethiopia, 2019

**Dimension D: attitude of community:** seventy six percent of key informants believed the presence of barriers. The most commonly quoted barriers were long distance to the health facility (87.5%), lack of road infrastructure (82.3%), cultural belief (76%), family influence (70.8%), and the inability of participants to afford the cost of attendance (70.8%) (Figure 4). The long distance to health facility was summed up by one key informant´s comment that: *“People who live in a place where far from the health facility feel that nothing could be done except home delivery”*. Several key informants stated that there was no lack of knowledge on the existence of programs. Another key informant mentioned that cultural norms were a barrier for people who were not comfortable *“giving birth at health facility”*. Programmatic barriers included a lack of site staff and trained professionals to give the services appropriately and lack of adequate funding. Barriers also included factors related to poor infrastructures (location of programs, geographical location, lack of finance, inadequate rest room and waiting home), weather concerns, and inaccessibility for people with disabilities. Fifty one percent of key informants agreed that their community would be supportive in promoting institutional delivery service programs. Fourty nine percent of key informants indicated that communities would not support for the programs. Despite the barriers, 54.2% of key informants said that there would be interest in new programs. Majority of the key informants (76%) said that there were conditions in which community members think that home delivery should be tolerated.

*“Some of the people who are not educated they say: My wife gave birth safely at home, if she delivers safely; they say why you are asking me to take her to health center” (Kebele administrators)*.

*“The most common beliefs held by the large community were; need to hear the sound of hen before going to health facility is, need to go health facility after animals get in their living room, need to go health facility after the sun shines and after the sun is raise to go health facility so that the women lose her energy” (Kebele security head)*

**Dimension E: resources:** majority of the key informants reported that the most commonly cited place of delivery is home with the help of Traditional Birth Attendant (TBA). We received few, but positive, responses when we asked about local business´ attitudes towards financially supporting those efforts. Approximately 98% of the key informants responded that their community had active volunteers to help in promoting health facility child birth service utilization but the volunteers need incentives.

*“There is no as such resource allocation and I could score low i.e. 1/10. Most of the community members did not need to allocate enough resource including time to stay at health facility at least for a week in health facility (female HAD leaders)”*.

*“The community did not voluntarily been contributed resources for maternal waiting, transportation and the like. The community members did not actively participate in the allocation of resource for the prevention of home delivery; even they did not have an idea on the resource allocation for such activity. They are only expecting everything from outside and externalizing. The volunteers expected some incentives from the above part of the community (Community leaders) (HEWs)*

*“The health development army was working by having good will in the future from the government. And it has to be supported by the government structure, needs advising, training and supportive supervision periodically” (Head of kebele female affairs)*.

**Preliminary proposed activities to improve readiness for the promotion of institutional delivery:** the CRM conveyed stage specific proposed activities to increase readiness to the next stage that was intended to keep the community on track for addressing the health problem in a more culturally acceptable way. Table 3 comprised the scores for each dimension in a descriptive interpretation, concentrated on the readiness scores gained from this study, as well as suggested undertakings to change the community to a higher stage of readiness. These proposed activities were comprehensive to allow the community and invested groups of participants the opportunity to determine and implement specific activities.

**Table 3:** proposed activities to increase the readiness of the community for the promotion of institutional delivery in Central Gondar Zone, Northwest Ethiopia, 2019

Dimension Stage	Score/Stage	Stage Definition	Goal to Maintain/Improve stage	Activities to Increase Stage
knowledge of community	6.67/Initiation	At least certain members of the community have knowledge regarding its occurrence and effects locally.	Provide community-specific information	Arranging staged matched capacity building for health professionals and non-health professionals begin some basic evaluation efforts.
Community effort	5/Preparation	At least certain members of the community are participating in developing and improving efforts.	Plan more specific strategies by collecting current evidences	Seek out local data sources about institutional delivery; supporter a community health event to boost offs the effort, bearing public meetings to develop tactics from the local level.
Leadership	4/Preplanning	At least certain of the leadership believe the problem is a worry in the community and certain type of effort is needed to solve it.	Increase awareness with concrete ideas	Review existing efforts in community to adjust the target inhabitant and reflect the level of achievement of the efforts.
Attitude of community	4/Preplanning	Certain members of the community believe as the issue is a worry in the community and certain type of effort is needed to it.	Increase awareness with concrete ideas	Introduce information about institutional delivery through presentations, local media and use different mass-media for public service announcements
Resource	3.73/Vague awareness	There are some resources (such as volunteers and local professionals) could be used for further efforts. There is little or no action to allocate these resources to this issue.	Increase awareness which the community can do somewhat	Get on the agendas and present information on institutional delivery using different events at local level and to other unconnected groups in the community, begin to initiate your own community health events and use those opportunities to also present information on institutional delivery.

## Discussion

Community-based interventions use community assessments as starting points for intervention activities [15, 24]. Community readiness assessment model is not an objective concept rather it is a subjective construct. The scoring system of CRM is performed via allotting values to ease comparison; though, qualitative data were collected as per the protocol [23, 25]. Throughout the interview period the CRM capture a community picture; a community, yet, is continuously shifting and level of readiness would be in change. The community score for each domain of the readiness was scored using the CRM working protocol; we have strictly following the steps provided on the model. In the process of determining the readiness level of each community by dimension and by overall stage conducting an assessment of the readiness level of the community is the key strategy. A score for a certain stage is given if and only if the entire statement is true; we have following all the necessary steps to score each domain of readiness. Each dimension was receiving a score for the readiness of the community. Thus, each dimension can be at a different readiness level and each score has a given meaning of readiness stages.

The score given in the assessment section introduce scales which we use to measure each dimension´s readiness level i.e. 1 of 9 level of readiness, starting from no awareness to great level of community ownership for the five dimensions using the assessment guidelines. The scores have its own statement which could help for leveling. The present study, assessing the readiness of the community to promote institutional delivery service utilization/ home delivery prevention/ in the study area is the first to apply the CRM. The overall readiness stage achieved for the promotion of institutional delivery (4.6) was the “Preplanning Stage”. At the “Preplanning Stage” the CRM suggests the community has some active leaders who are trying to plan efforts to which the community offers modest support. The qualitative analysis supports the interpretation of the lower readiness stage of the community for the promotion of institutional delivery service utilization, this could be due to the limited community engagement initiatives operating beyond the health facility setting and most of the community considers promoting institutional delivery for the large community is only the task of health systems and female HDA for the benefit of HEW and female HDA.

Higher community engagement will help the community to have a culture of institutional delivery in continuous way. The highest scoring dimension was: ‘knowledge of the community regarding the health problem´, which suggests the community has some knowledge about how much it occurs locally and its effect on the community. As stated by the authors of the CRM, the readiness should be at nearly the same level for each dimension before overall efforts can be effective [26]. Efforts therefore should be focused initially on the lowest scoring dimensions. Given the readiness scores for each dimension, addressing the ‘resources´ dimension, ‘leadership´, and the ‘attitude of community on the issue´ dimension would be the most appropriate initial targets for intervention because these represent the lowest readiness scores. This implies there should be enough allocation of resource for the promotion of health facility child birth service utilization in sustainable way, and also strengthening the commitments of local leader in the mobilization of the large community to have a favorable attitude towards the issue.

The transcripts of CRM providing all the necessary information which helps to design appropriate interventions for the issue going to be addressed [27]. The present study showed 53.3% of the community overall level of readiness was at the stage of vague awareness (stage-3) of the issue which is lower than a study conducted in Kansas, North America on the readiness level of the community for changing [28]. This might be due to the difference with the type of the issue to be address. Since the present study was accompanied within a strong application process of the issue of institutional delivery service utilization, social desirability bias might be reflected from the response. Community activities and commitments to the promotion of institutional delivery might be overstated by the participants with the hope of securing resources. Furthermore, the consensus process of the scorers aims to attenuate the transcript scoring process demands interpretive preference. The low score (3.73), vague awareness, for the resource allocation in the present study may indicate limited community resource allocation [26]. There are some resources (such as volunteers and local professionals) which could be used for further efforts. The lack of resources, (i.e. sustainable resource allocation) and resource mobilizations (funding highlights) the need for community members to be encouraged to take ownership of initiatives therefore when funding is stopped they can build on the commitment and find ways to continue, for example through income generation. This recommendation is in line with a research conducted in East mid lands of UK [29]. Resources are also required within this community to enhance the mobilization skills of HDA and the community at large, who may lack enthusiasm [29].

There is little or no action to allocate these resources to the specified issue. Which implies there has to be better resource allocation for the prevention of home delivery/promotion of institutional delivery/ by the large community. In contrast, the mean score for community knowledge and existing efforts of the community was the highest of the dimensions. The difference between these scores indicates efforts existed and communities could have been informed of them; the limited resource allocation suggests a communication gap between the community and the people involved in home delivery prevention efforts. This implies the need to strengthening the efforts of the community in the promotion of health facility child birth service utilization and creating sense of ownership in the allocation of enough resource for the issue. The communities´ low stage of readiness indicates the need for more health facility child birth service utilization programming is recognized locally but the current efforts are not focused or detailed, and leadership and motivation are minimal. The key informants recognized the benefits of institutional delivery but felt further education about these benefits was needed. Although community-based programs were planned for the community, the key informants felt their communities would support such programs.

The variability among the leadership scores indicated differences in community leaders´ prioritization of home delivery prevention/promotion of institutional delivery among competing project; for this reason, low leadership scores (4) were an indicator for the intervention to be involved. The higher prevalence of home delivery in Central Gondar Zone could be attributed to lack of transportation, low-income, political instability and poor awareness of the residents. Conferring to the CRM, strategies for communities in the vague awareness stage should be aimed at raising awareness, empowering communities to make changes, and soliciting community support [23]. Potential strategies could include presenting information at local events regarding the benefits of institutional delivery service utilization, the location of current programs, resources for increasing programming (eg, funding opportunities, training) and conducing level matched educational intervention on the issue. Other strategies include distributing flyers and posters; creating billboard advertisements; conducting local surveys and sharing survey data with communities; and publicizing home delivery prevention activity benefits and opportunities through the local journalism.

Communities with a high overall score (eg, a score of 6.67 for knowledge of the community regarding the health problem, corresponding with the initiation stage of readiness) may already have sufficient motivation and momentum to initiate and sustain intervention components on their own [16]. This implies adequate evidence has to be gathered to justify initiation of efforts, and activities are underway. Communities with a low overall score (eg, a score of 3.73 for resource allocation, corresponding with vague awareness) would need to dedicate significant efforts to raising awareness and building relationships in advance of implementing any intervention components in order to do something in the resource allocation for the activity. This suggests furthermost sense; there may be an inhabitant concern, however there is no prompt inspiration or willingness to do whatsoever about it. Therefore, present information on institutional delivery at local community events and to unrelated community groups, begin to initiate your own community health events (pot lucks, potlatches, etc.) and use those opportunities to present information on institutional delivery. The qualitative information gained from the interviews enhanced the understanding of the communities.

To increase the readiness level of the community to promote institutional delivery in the study area, it is recommended that efforts shall be made to boost awareness of the community at large on health service utilization behavior and therefore the prevalence of institutional delivery and allocation of enough resource in sustainable way. To attain sustainable initiatives, communication and collaboration between the community, leadership and medical experts should aim to incite community engagement and ownership over initiatives. This survey identified barriers and culturally specific issues that might enhance program adoption. In order to achieve a success in the adoption of the program the key respondent´s recommended locally acceptable, accessible and free cost packages of activities. Evidence derived from our survey is accustomed level match intervention strategies for promoting institutional delivery´ to communities in Central Gondar zone in line with their level of readiness.

**Limitations of the study:** the model cannot prescribe the small print of exactly what to try to do or to fund in each community. The context across communities differs and prevention strategies must be tailored to make sure appropriate fit. Establishing the validity for the readiness measure of the community is challenging within the absence of a real readiness value that would be captured through an objective protocol.

## Conclusion

The promotion of institutional delivery/prevention of home delivery/has not previously been assessed through the CRM, and particularly in a rural setting of developing country. Because of the massive number of home delivery and also the high level of urgency to handle home delivery in Ethiopia, it´s undoubtedly essential to integrate the community viewpoints and attitudes in these discussions and to verify how they´re investing in resolving home delivery. The present study provided baseline readiness scores of community for the promotion of institutional delivery within the community of Central Gondar zone in Ethiopia. In next steps during this initiative, we are going to develop and implement sustainable, community and culturally specific strategies supported these baseline readiness stages. Additionally to a spotlight on reducing home delivery and replaced by institutional delivery, future plans should also include a reassessment of readiness after community interventions occur. Moreover, continuous monitoring and evaluation of whether these public health issues begin to resolve locally are going to be a very important outcome measure for the community?

### What is known about this topic

- Low institutional delivery service utilization;
- Making child birth at health facility ensures safe child birth and reduces complications and maternal death;
- Evaluating the readiness level of the community in the participation of promotional event was crucial step to design an intervention plan.

### What this study adds

- Over all community level of readiness on the promotion of institutional delivery service utilization;
- Determining the community level of readiness on the promotion of institutional delivery service utilization in five dimension;
- Stage matched intervention were designed.
